# TRAIL Dependent Fratricidal Killing of gp120 Primed Hepatocytes by HCV Core Expressing Hepatocytes

**DOI:** 10.1371/journal.pone.0027171

**Published:** 2011-11-14

**Authors:** Stacey A. Rizza, Kishore B. Challagundla, Sekar Natesampillai, Gary D. Bren, Jaromir Sykora, Henning Walczak, Andrew D. Badley

**Affiliations:** Division of Infectious Diseases, Mayo Clinic, Rochester, Minnesota, United States of America; University of Montreal, Canada

## Abstract

The mechanism by which HIV and HCV cooperatively accelerate hepatocyte damage is not clearly understood; however, each virus affects the TRAIL: TRAIL- receptor system. We, therefore, questioned whether the independent effects of HCV and HIV combine to synergistically result in TRAIL dependent hepatocyte killing. We describe that Huh7 hepatocytes treated with HIV gp120 results in both increase TRAIL-R2 expression and an acquired sensitivity to TRAIL mediated killing. Moreover HCV infection and HCV core expression alone in Huh7 cells upregulates TRAIL. Co-incubation of HIV gp120 primed hepatocytes with HCV core expressing hepatocytes results in the selective death of the HIV gp120 primed hepatocytes that is selectively blocked by TRAIL–R2-Fc fusion protein. Liver biopsies from HIV mono-infected patients have increased TRAIL-R2; biopsies from HCV infected patients have increased TRAIL, while co-infected liver biopsies have increased PARP cleavage within hepatocytes indicating enhanced apoptosis. These findings suggest a pathogenic model to understand why HIV/HCV co-infection accelerates liver injury.

## Introduction

HCV and HIV infections are frequent worldwide; in the USA more than four million people are infected with HCV, which can cause liver fibrosis, cirrhosis, end-stage liver disease, and hepatocellular carcinoma. Approximately 30% of HIV- infected individuals are co-infected with Hepatitis C Virus (HCV) due to their similar modes of transmission [1], as evidenced by the fact that 80% of intravenous drug users (IVDU) infected with HIV are co-infected with HCV. HIV/HCV co-infection results in an increase in morbidity and mortality than occurs with either HIV or HCV mono-infection [2], [3].

The pathologic mechanism(s) underlying the accelerated liver disease associated with HCV during HIV co-infection are not clearly understood, but have been proposed to be due to a loss of HCV-specific CD4+ T cells[4]. Such a model accounts for improvements in liver disease which occurs after highly active antiretroviral therapy (HAART)-induced immune reconstitution [5], and lower CD4+ T cell counts being associated with worsened liver function [6]. Alternative models include HIV infection altering the host cytokine response to HCV [7], enhancing the ability of HCV to infect target cells [8], or HIV exacerbating the cytotoxicity of HCV [9]. It also has been suggested that HIV and HCV may cooperatively induce hepatocyte injury through apoptosis [10]. Hepatocyte apoptosis in turn leads to liver fibrogenesis and ultimately cirrhosis [11].

Apoptosis contributes to many liver diseases, including viral hepatitis. Activation of the TNF super-family of death inducing receptors results in hepatocyte apoptosis in several models of liver disease [12]. Hepatocytes express Fas (CD95), TRAIL-R1 and –R2 (TNF-related apoptosis-inducing ligand receptor -1 and -2) (DR5/CD262) and TNF-R1 (TNF receptor type 1) on the cell surface [13], [14], [15]. After ligation by their respective ligands, these death receptors aggregate and form the death inducing signaling complex (DISC) which causes caspase 8 activation that ultimately results in mitochondrial membrane permeabilization, formation of the apoptosome, which in turn activates effector proteases and nucleases, resulting in the phenotypic changes of apoptosis [16].

There is increasing evidence that liver damage caused by HCV is due to hepatocyte apoptosis, with more hepatocyte apoptosis being present in livers from HCV-infected compared to non-infected individuals [17], [18]. Using antibodies specific for active caspase-3 and -7, as well as the cleaved form of PARP, caspase activation and PARP cleavage is increased in livers infected with HCV compared to non-infected livers and caspase activation, as well as hepatocyte apoptosis are directly correlated with the grade of necro-inflammatory injury (reviewed in [18], [19]). Hepatocyte apoptosis may be caused by both immune mechanisms and direct cytopathic effects of HCV [20]. Fas (CD95) has been the most thoroughly investigated death inducing ligand during HCV infection. Fas is expressed on hepatocytes during HCV infection and levels correlate with the severity of liver inflammation. Cell surface associated Fas and FasL levels are also associated with increased hepatocyte apoptosis during HIV/HCV infection [15], [17]. Of note, several recent reports indicate that hepatocytes obtained from patients with HCV also have greater TRAIL expression than hepatocytes from uninfected controls [21], [22].

We have reported that X4 HIV gp120 envelope protein binding to CXCR4 on the surface of human hepatocytes activates JNK II, causing a transcriptional up regulation of the TRAIL receptor, TRAIL-R2 (DR5/CD262), which also is associated with an acquired state of TRAIL sensitivity [14]. More recently this observation has been extended to JFH1 HCV infected Huh 7.5.1 cells, where inactivated HIV causes upregulation of TRAIL-R2, and initiates a TRAIL dependent apoptosis [23] Altogether, such data support a model wherein HIV enhances TRAIL-R2 expression and TRAIL sensitivity, while an unknown factor associated with HCV infection promotes TRAIL expression and function. The purpose of the current report is to discover which factor associated with HCV causes hepatocyte expression of TRAIL, and to determine whether these changes result in the autocrine or paracrine death of HCV expressing, or HIV treated cells, and to determine whether the changes which are occur *in vitro* are also seen *in vivo*.

## Results

### X4 HIV Gp120 Increases TRAIL-R2 but not TRAIL on Huh7 cells

**Figure 1:**
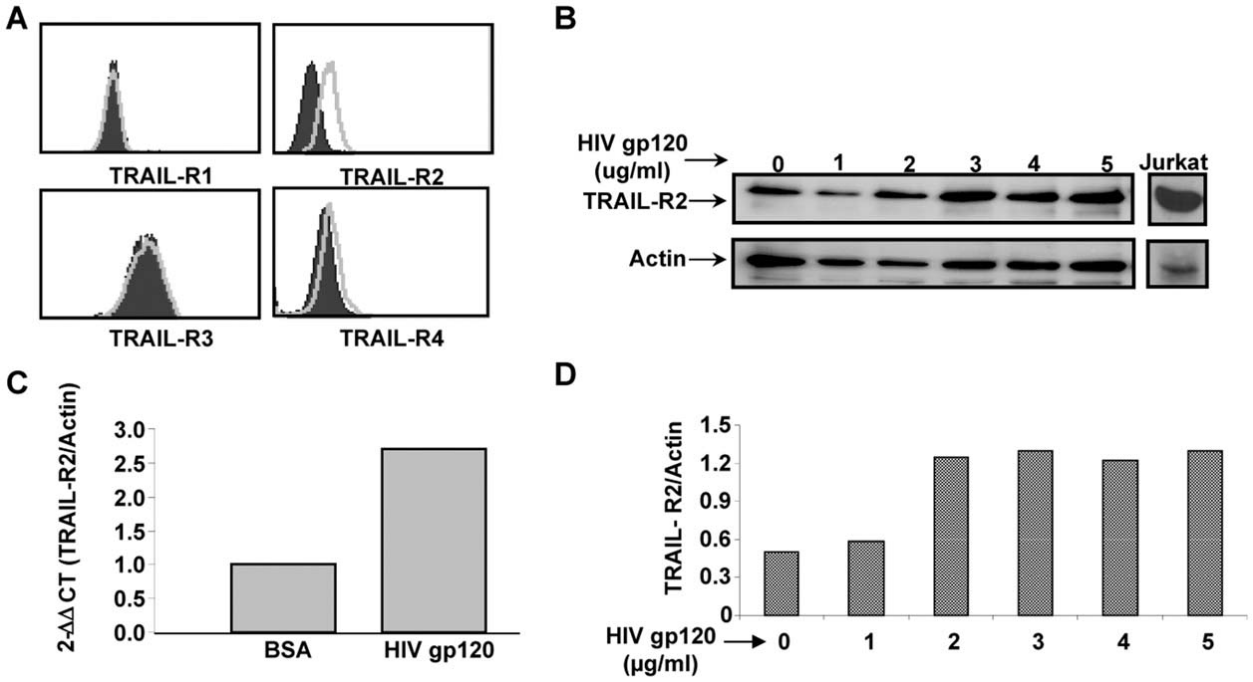
HIV gp120 Increases TRAIL-R2. (A) Huh7 cells were incubated with increasing concentrations of HIV gp120 and analyzed for TRAIL-Receptor expression by Flow cytometry for TRAIL-R1, -R2, -R3, -R4 (BSA  =  black, HIV gp120 =  Grey), (B and D) Western blot (C) or real time PCR.

**Figure 2:**
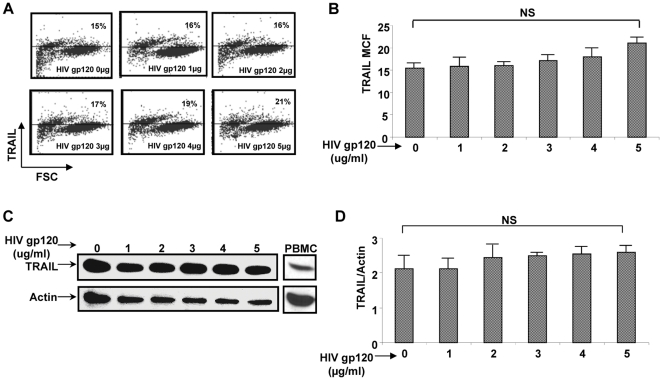
HIV gp120 Does not Increase TRAIL. (A and B) Huh7 cells were incubated with increasing concentrations of HIV gp120 and analyzed for TRAIL expression by Flow cytometry (C and D) and Western blot.

It is established that HIV increases TRAIL-R2 expression on hepatocytes, renders the cell sensitive to TRAIL, and independently induces a low degree of hepatocyte apoptosis [14], [24]. Therefore, we questioned whether HIV gp120 also increases TRAIL expression, which then might cause the observed hepatocyte apoptosis. As we have previously demonstrated, there was greater TRAIL-R2 expression, but not TRAIL-R1, -R3 nor –R4 when Huh7 cells were incubated with increasing concentrations of HIV gp120 (Fig. 1A and B)[14]. This is confirmed by real time PCR (Fig. 1C and D). However, increasing concentrations of HIV gp120 did not alter TRAIL expression on Huh7 cells by flow cytometry (Fig. 2A) or Western blot (Fig. 2C). Therefore, HIV gp120 increases TRAIL-R2 expression on Huh7 cells, but does not alter TRAIL expression. Although the Huh7 cell is TRAIL sensitive after HIV gp120 treatment, there is little spontaneous death of the hepatocyte without the addition of exogenous TRAIL [14].

### HCV Core Protein Increases TRAIL but not TRAIL-R2 on Huh7 cells

**Figure 3:**
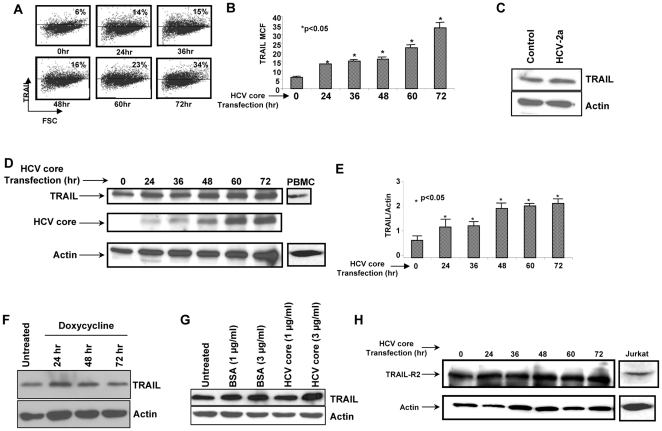
HCV core protein Increases TRAIL but not TRAIL-R2. (A and B) Huh7 cells were transfected with HCV core protein for increasing amounts of time and then analyzed for TRAIL expression by Flow cytometry or (D and E) Western blot. (C) Huh7.5 cells were infected with the JFH1 HCV genotype 2a and analyzed for TRAIL by Western blot. (F) Huh7 cells were treated with doxycycline for increasing amounts of time and analyzed for TRAIL by Western blot (G) Huh7 cells were treated with increasing concentrations of soluble HCV core protein or control and analyzed for TRAIL by Western blot (H) In parallel HCV core transfected Huh7 cells were analyzed for TRAIL-R2 expression by Western blot.

It has been previously observed that HCV infection in the JFH1 HCV model using Huh7.5.1 cells results in enhanced TRAIL expression [23], and a similar effect has been reported to occur after HCV infection of livers *in vivo* [21], [22], [25]. Since it remains unknown what factor (s) associated with HCV are necessary or sufficient for this effect, we questioned whether individual HCV protein(s) could induce TRAIL expression in hepatocytes. Ultimately we focused on the HCV core protein since it is highly conserved and has been associated with several immunomodulatory functions [26], [27], including modulation of apoptosis signaling [28], [29]. Huh7 cells were transfected with HCV genotype 1b core protein, as this represents the most common HCV genotype in North America and Western Europe, and patients with this virus have accelerated liver disease [30]. Cells expressing HCV core had increased TRAIL surface expression compared to control, as determined by flow cytometry (Fig. 3A and B) and western blot (Fig. 3D and E). A similar increase in TRAIL was seen following HIV infection using JFH1 HCV in Huh7.5.1 cells (Fig. 3C). The HCV core protein expression system is doxycycline responsive, however, doxycycline alone did not change TRAIL levels (Fig. 3F). The increase in TRAIL only occurred when HCV core protein was intracellularly expressed and not by extracellular soluble HCV core protein (Fig. 3G). Conversely, HCV core expression in Huh7 cells did not increase TRAIL-R2 expression (Fig. 3H), demonstrating that HIV and HCV exert complementary effects on the hepatocyte; HCV core protein increases TRAIL, but not TRAIL-R2 while HIVgp120 increases TRAIL-R2 and confers an acquired state of TRAIL sensitivity, while not impacting TRAIL levels.

### HIV increases TRAIL-R2 and HCV Increases TRAIL *in vivo*

**Figure 4:**
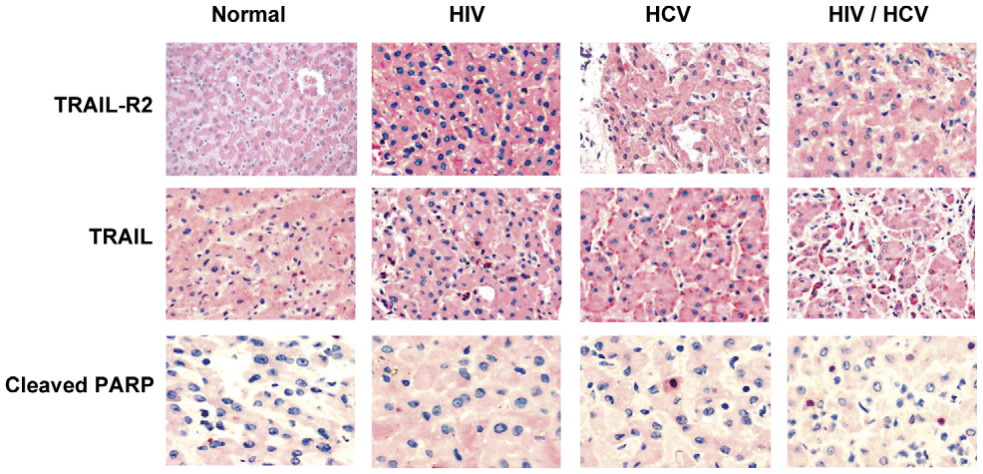
*In vivo* analysis of TRAIL, TRAIL-R2 and Apoptosis in Human Livers. Liver samples from 10 HIV infected, 10 HCV infected, 10 HIV/HCV infected or 10 uninfected individuals were stained by Immunohistochemistry for TRAIL, TRAIL-R2 or cleaved PARP. The images were taken with an Olympus Microscope with the 20x objective (TRAIL, TRAIL-R2), and 40x objective (PARP). Representative samples are shown.

In our model, HIV gp120 treatment increases TRAIL-R2 expression on Huh7 cells, and HCV core protein increases TRAIL expression. In order to confirm that these changes also occur *in vivo*, liver samples from 10 uninfected individuals, 10 HIV infected, 10 HCV infected and 10 HIV/HCV co-infected individuals were stained by immunohistochemistry for TRAIL-R2, TRAIL and cleaved PARP as a measure of apoptosis (Fig. 4). Normal liver samples taken from individuals not infected with HIV or HCV have minimal TRAIL-R2, and minimal TRAIL expression. TRAIL-R2 levels are increased in liver samples from HIV-infected individuals and TRAIL staining is increased in HCV infected livers. Liver samples from HIV/HCV co-infected individuals have increased TRAIL and TRAIL-R2 staining (Fig. 4). Furthermore, HIV/HCV co-infected livers have greater hepatocyte apoptosis, as determined by staining of cleaved PARP, than HIV mono-infected, HCV mono-infected or uninfected livers, consistent with previous reports[17], [31].

### Co-incubation of HIV and HCV Treated Huh7 cells Results in a TRAIL Mediated Apoptosis

**Figure 5:**
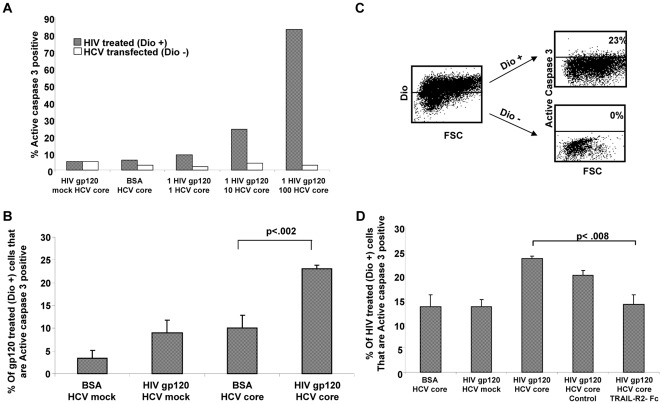
Co-incubation of HIV/HCV treated hepatocytes kills HIV treated hepatocytes via TRAIL. (A) Huh7 cells expressing HCV core were co-incubated with HIV gp120 treated hepatocytes at increasing effector-to-target ratios, and cell death of HIV treated (Dio positive) hepatocytes were determined by flow cytometry for active caspase 3 expression. (B) HIV gp120 or BSA control treated cells were incubated with HCV core or mock transfected cells and death analyzed in the gp120 or BSA treated cells. (C and D) HIV gp120 or BSA treated cells were incubated with HCV core or mock transfected cells, in the presence or absence of a TRAIL-R2-Fc fusion protein, or control-Fc fusion protein, and cell death analyzed in the gp120 or BSA treated cells.

Our observation of increased TRAIL-R2 expression and enhanced TRAIL sensitivity induced by HIV and increased TRAIL expression induced by HCV core, predicts that the presence of both viruses should result in augmented hepatocyte death. To test this hypothesis, Huh7 cells were stained with the lipophilic dye Dio, treated with HIV gp120 and then co-incubated at increasing effector-to-target ratios with HCV core protein transfected Huh7 cells. Following an overnight incubation, cells were analyzed by flow cytometry for active-caspase-3 expression to determine the levels of apoptosis in the Dio positive (HIV gp120 treated) and Dio negative (HCV core expressing) cells. There was no Dio leak between hepatocytes (data not shown). HIV core expressing (Dio positive) target cells had greater apoptosis than HCV treated hepatocytes and this effect increased with increasing E:T ratios (Fig. 5A). The effect was not present when target cells were treated with control protein rather than HIV gp120 (Fig. 5B). The HCV core expressing (Dio negative) effector cells had no greater apoptosis than untreated cells (data not shown). Pre-incubating the Huh7 cells with the TRAIL-R2-Fc fusion protein, blocked the increased apoptosis that resulted from co-incubating HIV and HCV treated hepatocytes (Fig. 5C and 5D). These data demonstrate that HIV gp120 increases TRAIL-R2 expression and TRAIL confers sensitivity in treated hepatocytes; HCV core protein increases TRAIL expression in hepatocytes and co-incubating the HIV gp120 and HCV core expressing liver cells results in an accelerated TRAIL mediated apoptosis, selectively in the HIV treated cells. The relevance of this *in vivo* is suggested by similar changes being observed in liver biopsies from patients with HIV infection, HCV infection or both.

## Discussion

It is clear that HIV worsens the course of HCV infection by increasing hepatocyte apoptosis, liver fibrosis and inflammation and quickening the progression to end stage liver disease [6]. It remains unclear how HIV propagates HCV liver disease, but clinical evidence demonstrates that decreasing HIV viral replication and improving CD4+ T cell counts, improves markers of liver disease [5], [6].

HIV RNA and p24 antigen have been isolated from liver samples, presumably from HIV infected immune cells, indicating that HIV proteins are in close proximity to hepatocytes during HIV infection [32], [33]. These proteins also contribute to the depletion of CD4+ T cells through multiple mechanisms, including HIV gp120 mediated bystander killing of uninfected cells [34]. HIV gp120 also induces death of non-immune cells, including neurons and hepatocytes [10], [24], [35], [36]. Therefore, HIV gp120 causes at least two distinct effects upon the liver: 1) low level of background death [24] which may account for the increased liver disease in HIV mono-infected patients compared to uninfected patients, and 2) increasing TRAIL-R2 expression, and co-incident acquired sensitivity to TRAIL mediated apoptosis [14], which likely contributes to the accelerated course of co-morbid liver disease during HCV co-infection. 

Within our proposed model, one would expect increased hepatocyte apoptosis during any disease states in which TRAIL and TRAIL-R2 are increased. In addition to HIV gp120, a number of other stimuli can increase TRAIL-R2 expression or TRAIL sensitivity in hepatocytes. The Hepatitis B virus protein MHBs(t) sensitizes human hepatocytes to TRAIL [37] and is mediated via Bax signaling [38]. Free fatty acid induced steatosis causes TRAIL sensitivity in human hepatocytes [39] which is exacerbated by treatment with alcohol [22]. In addition, bile acids increase TRAIL-R2 and induce TRAIL sensitivity in human hepatocytes. This process is mediated via a c-Jun N-terminal kinase-dependent pathway involving Sp1 [12]. Medications can also sensitize cells to TRAIL mediated apoptosis and are being investigated as a means to treat otherwise TRAIL resistant cancers. Doxorubicin treatment, for instance, sensitizes glioblastoma cells [40] and liver cancer cells to TRAIL via the BH3-only proteins Bim and Bid [41]. At supra-therapeutic levels, even HIV protease inhibitors, such as Nelfinavir, can sensitize cells to TRAIL [42]. This may in part explain why there is worse liver disease during Hepatitis C Virus infection and any of these co-existing conditions.

Several reports indicate that hepatocytes obtained from patients with HCV have increased TRAIL expression compared to hepatocytes from uninfected controls [21], [22]. Our data expands upon these observations with the novel findings that this effect localizes to the HCV core protein. Moreover, we now report that increased apoptosis occurs in hepatocytes when HCV core expressing hepatocytes produce TRAIL which induces fratricidal killing of HIV gp120 primed hepatocytes that manifest both increased TRAIL-R2 as well as enhanced TRAIL sensitivity. This model is further supported by immunohistochemistry in tissues from HIV or HCV mono-infection, or HIV/HCV co-infection where similar changes occur *in vivo* as what we observed *in vitro*.

While we demonstrate that HCV core protein alone is sufficient to increase TRAIL expression, it is possible that other HCV structural or non-structural proteins may also alter TRAIL expression. Furthermore, our model does not exclude the possibility that HCV infection triggers an indirect effect that in turn regulates TRAIL expression. HCV infection results in a number of alterations in the immune system most notably, HCV causes a Type 1 interferon response. Type 1 interferon induces TRAIL expression on NK cells which is associated with control of HCV infection in patients [43]. Furthermore, in a cell culture model, the HCV isolate JFH-1 resulted in an interferon response which caused apoptosis of infected cells [44]. Interestingly, amino acid substitutions in the HCV core protein during clinical HCV infections, alters the response to interferon based HCV therapy [45], suggesting that HCV core plays a role in interferon mediated effects and possibly TRAIL mediated apoptosis.

We propose a model of HIV/HCV mediate liver disease whereby co-incubation of HIV gp120 treated and HCV core expressing hepatocytes results in accelerated hepatocyte death which is TRAIL mediated. Therefore, untreated HIV infection would create a TRAIL sensitive hepatocyte with increased TRAIL-R2 expression. When a second insult is introduced into the liver, such as HCV infection, TRAIL production increases and there is TRAIL mediated hepatocyte death; importantly TRAIL antagonists reverse this process, suggesting a novel treatment strategy for liver disease associated with HIV/HCV co-infection.

## Methods

### Chemicals and Reagents

The following chemicals and reagents were used for experiments in the manuscript: Dulbecco's modification of Eagle's medium (DMEM) (Mediatech Inc., Manassas, VA), Fetal bovine serum (FBS) (Atlanta Biologicals Inc., Lawrenceville, GA), Recombinant superkiller TRAIL (skTRAIL) (Alexis Biochemicals, San Diego, CA), Recombinant HIV-1 IIIB envelope glycoprotein 120 (gp120) and recombinant human T- cell receptor sCD4 (Immuno Diagnostics Inc., Woburn, MA), Bovine serum albumin (BSA) (Sigma Chemical Co, St. Louis, MO), anti-actin and anti-tubulin antibodies (Molecular Probes, Carlsbad, CA), Antibodies to TRAIL-R1, -R2, -R3, -R4 and isotype control, for flow cytometry (BD Bioscience, San Diego, CA) and TRAIL-R2 for Western blot (Capralogics Inc., Hardwick, MA), active caspase-3 (BD Biosciences), TRAIL-R2-Fc chimera (R & D system, Minneapolis, MN), His tagged soluble HCV core protein (PROSPEC, Ness Ziona, Israel).

### Cell Viability

The human hepatocyte cell line Huh7 (ATCC) was cultured in DMEM with 10% FBS and 100 units/mL of penicillin, 100 µg/mL streptomycin, and 200 mM glutamine. The Huh7 cells were plated at a density of 3×10^5^ cells/well in a 6 well plate. 5 µg/mL HIV glycoprotein 120 IIIB [46] was used to prime Huh7 cells for six hours, and then cells were incubated with 100 ng/mL of skTRAIL or with HCV core transfected Huh7 cells for thirty six hours. In some experiments, Huh7 cells were pre-incubated for 1 hour at 37 C with rhTRAIL-R2/Fc chimera (0.5 µg/ml) or control before co-incubation. Apoptosis was determined by staining for Active Caspase 3 and analyzing by flow cytometry, as described below.

### HCV Core Transfection

Huh7 cells were co-transfected with a mixture of supercoiled pTet-on, pTts (Clontech) and pTRE2-191 and lipofectamine 2000 reagent (Invitrogen, CA). Transfected cells were grown in complete culture medium for 24 h. To induce the conditional expression of HCV-core protein, transfected cells were treated with Doxycycline 1 µg/mL for the indicated period and the expression of core was confirmed by western blot analysis.

### Western Blots

Huh7 cells were treated with 5 µg HIV gp120 for 24 hours and lysed in lysis buffer (20 mM Tris, pH 7.5, 150 mM NaCl, 2 mM EDTA, 0.1% Triton-X100, 2 mg/ml aprotinin, 5 mg/ml leupeptin, 1 mM PMSF and 1 mM Saponin) on ice. Jurkat cells and PHA and IL2 stimulated PBMCs were used for positive controls. 30 µg of protein was resolved on a 15% sodium dodecyl sulfate polyacrylamide (SDS-PAGE) gel and electrotransferred to polyvinylidene fluoride (PVDF) membranes (Millipore Corporation, Bedford, MA). Membranes were blocked with 5% nonfat milk in TBST [Tris base (10 mM), NaCl (150 mM), Tween (0.1%), pH 7.5] for two hours at room temperature and probed with anti- TRAIL-R2 (1∶1000) antibodies overnight at 4 C. Actin (Sigma, St. Louis, MO) was used as a loading control and intensity was measured using EL Logic 2200 imaging software system.

### Real Time Quantitative PCR

Total RNA was isolated from control or GP120 (1 µg/ml) treated Huh7 cells for 48 h, using Qiagen RNeasy mini kit (Qiagen, Valencia, CA) and reverse transcribed using the ABI High Capacity cDNA Reverse Transcription Kit (Applied Biosystems) with 2.5 µM random oligos, and 1 µg of RNA. The cDNA was amplified in 25 µl of PCR buffer with Platinum SYBR Green qPCR SuperMix-UDG with ROX kit (Invitrogen, CA) in the presence of TRAIL-R2 or actin specific primers (sense primer for TRAIL-R2 5′-AAGACCCTTGTGCTCGTTGT - 3′, antisense primer 5′-AGGTGGACACAATCCCTCTG - 3′, actin sense primer 5′ -GAAACTACCTTCAACTCCATC - 3′) and antisense primer 5′ –CGAGGCCAGGATGGAGCCGCC -3′. The PCR conditionsincluded a hot-start by 95°C for 2 min followed by 40 cycles of 95°C for 15 s, 60°C for 1 min. Samples were run in triplicates on the ABI PRISM 7700HT sequences detection system (Applied Biosystems) to determine the threshold cycle (C_T_). To determine the variation on mRNA expression, we analysis according to the Delta-Delta threshold (Ct) method, each Ct value was first normalized to the respective beta-actin Ct value.

### Flow Cytometry

Huh7 cells were harvested, washed in cold PBS, and fixed in 2% paraformaldehyde overnight at 4 C. Cells were blocked with 2% BSA/PBS and stained with phycoerythrin (PE) conjugated anti-human anti-TRAIL-R1,-R2, -R3, -R4 or isotype control mAb (1∶40) or rabbit polyclonal anti-active caspase-3 antibodies (1∶40) and then permeabilized with 0.1% NP-40 for 15 minutes or isotype control (Abcam Inc., Cambridge, MA) for one hour on ice, and analyzed by flow cytometry (FACScan flow cytometer, BD Biosciences, San Diego, CA).

### Immunohistochemistry

With Mayo Clinic Institutional Review Board approval (IRB 1039-03), liver samples were obtained through the National Disease Research Interchange (NDRI, Philadelphia, PA) from 10 HIV infected, 10 HCV infected, 10 HIV/HCV infected and 10 uninfected individuals. All patients were HBV negative and died of unrelated traumatic causes. Anti-TRAIL and anti-TRAIL-R2 staining was performed in 10 mM citrate at pH 6.0 and 99 C for 20 min. To block non-specific antibody binding, sections were incubated with blocking solution 1 [PBS, BSA 20 mg/ml (Serva, Germany, Cat. No. 11924), human IgG 1 mg/ml Gamma-Venin, (Aventis Behring)] for 20 min. Sections were then incubated overnight at 4 C in blocking solution in the presence of the first antibody or isotype-matched antibodies IgG1 and IgG2b at the same concentration, both obtained from DakoCytomation GmbH. Sections were washed twice in PBS and incubated with blocking solution 2 [20% normal goat serum from Dianova GmbH (Jackson ImmunoResearch Laboratories, Inc.)] for 20 min. After blocking, sections were incubated with secondary biotinylated antibody for 30 min at room temperature, rinsed twice for 5 min in PBS and incubated for 30 min with streptavidin-alkaline phosphatase [Super-Sensitive Detection Kit, BioGenex San Ramon, CA (DCS Innovative Diagnostik-Systeme GmbH, Hamburg, Germany)]. Thereafter, sections were rinsed twice in PBS, incubated with fast red substrate (Fast Red Substrate System, DakoCytomation GmbH) and counterstained with haematoxylin (DakoCytomation GmbH).

### Statistical Analysis

All results were expressed as the mean standard deviation (SD) and repeated at least three times as indicated in the figures. Statistical comparisons were made by student's *t* test employed to calculate the significance between the paired observations. A P value of 0.05 was used as the level of significance.
